# A reassessment on the state of knowledge of Chilean Falconidae in the last hundred years

**DOI:** 10.3897/zookeys.642.9877

**Published:** 2017-01-03

**Authors:** Ricardo Soto-Saravia, Víctor Hugo Ruiz, Alfonso Benítez-Mora, Margarita Marchant, Emmanuel Vega-Román

**Affiliations:** 1 Programa de Doctorado en Sistemática y Biodiversidad, Departamento de Zoología, Facultad de Ciencias Naturales y Oceanográficas, Universidad de Concepción. Casilla 160-C, Concepción, Chile; 2 Departamento de Zoología, Facultad de Ciencias Naturales y Oceanográficas, Universidad de Concepción. Concepción, Chile. Barrio Universitario s/n Concepción, Chile; 3 Centro de Investigación en Recursos Naturales y Sustentabilidad, Universidad Bernardo OHiggins, Fábrica 1990, Santiago, Chile; 4 Programa de Magíster en Enseñanza de las Ciencias, Universidad del Bío-Bío, Chillán, Chile

**Keywords:** Birds of prey, Falconidae, Knowledge, Diversity, Chile, Natural history

## Abstract

Eight species of falcons (Falconidae) have been recorded in Chile. To date, all relevant studies considered birds of prey in general, with no specific focus on this family. Based on a comprehensive review of the literature, an updated report is presented on the state of knowledge of falcons in Chile. This data set comprises a total of 165 studies published from 1915 to 2015. Scientific productivity was lowest in 1945-1955 and highest in 2005-2015, with a steady increase since 1985. However, the focus of research in Chile is biased towards two species: *Milvago chimango* and *Falco sparverius*. Two administrative regions, Santiago Metropolitan Region and Araucanía, were the most studied whereas Arica, Tarapacá, and Antofagasta regions accounted for fewer than 1% of the studies. Faunistic studies (including abundance) were the most common research topic. It is suggested that the lack of knowledge regarding species in the genus *Phalcoboenus* may negatively affect the conservation status of these species, and believed that the lack of preference for certain research topics, such as systematics and natural history, are the result of historical factors including the decrease of field biology and perhaps a biased interest of the researchers. Finally, this review highlights the paucity of information on falcons and provides a framework for directing future research.

## Introduction

Falcons (family Falconidae) are small to medium sized, exclusively diurnal birds of prey, which are top predators inhabiting a broad range of habitats (Yáñez et al. 1982, Biondi et al. 2005). Falcons are considered good indicators of ecological and environmental health (Rau and Jaksic 2004), as are other birds of prey (Pfeiffer and Meyburg 2015). They fulfil an important role maintaining control over plague species and regulating the ecosystem (Rau 2014). The 64 species are grouped in 11 genera and 2 subfamilies: Polyborinae (caracaras and forest falcons) and Falconinae (true falcons and falconets) (White et al. 1994). Eight species of falcons inhabit Chile: five species of caracaras (*Caracara plancus* (Miller 1777), *Milvago chimango* (Vieillot 1816), *Phalcoboenus albogularis* (Gould 1837), *Phalcoboenus australis* (Gmelin 1788), and *Phalcoboenus megalopterus* (Meyen 1834)), and three Falconinae (*Falco femoralis* (Temmnick 1822), *Falco peregrinus* (Tunstall 1771), and *Falco sparverius* (Linnaeus 1758)).

Research and reviews in Chile have mostly focused on birds of prey in general. A review was carried out by Muñoz-Pedreros and Norambuena (2011), who covered a period of 200 years, and focused on publication type, temporal trends, and research topics. Although only covering 30 years of scientific research, Raimilla et al. (2012) identified a marked bias towards nocturnal species, centering on diet and reproductive aspects. Two other reviews focused on one type of bird of prey. Figueroa et al. (2015) focused on the biology of owls in Chile and relevant conservation strategies. Figueroa (2015) documented a lack of research on the natural history of *Milvago chimango*. All these investigations are significant as they expose a deficiency in information, and thus, indicate where more research is warranted, regardless of possible limitations typical to this type of studies as indicated by Bimrose et al. (2005). Limitations may include a limited temporal scope, insufficient access to information, and the selection and classification of studies.

Our aim is to describe the current state of knowledge regarding falcons in Chile based on a comprehensive review of the literature. We identify deficiencies in temporal, thematic, geographic, and species-specific knowledge, discuss the possible causes and suggest directions for future research.

## Methods

The bibliographical review consisted of an exhaustive search of the ornithological literature on falcons in Chile, published between 1915 and 2015, and presented in scientific publications, national and international journals (both indexed and non-indexed), books, book chapters, and undergraduate and graduate theses (only those with online free access). Relative productivity was measured and compared across time (10 year intervals beginning in 1915), space (administrative regions), subject (research topic), and species.

We classified research topics into 13 categories identified by Muñoz-Pedreros and Norambuena (2011). However, due to the lack of literature for some topics, only 11 applied to our data set: Natural History (NH): studies that cover descriptions of morphology, distribution, identification, ecology, and systematics. Field manuals and monographs also fall into this category; Systematics and Taxonomy (ST): studies on classification, description of new species, species reviews, and phylogenetic analyses; Distribution and Biogeography (DB): studies focusing on distribution patterns, updates in distribution ranges, and the study of processes which originate and modify said distribution; Faunistics, Biodiversity, and Abundance (FBA): studies with observation data, new registries, sightings, species diversity, and abundance data. Studies that present abundance data are considered in this category, as ecologic studies contemplate gathering said data along with visual observations and registries, which coincide with the most common methodologies (see: Bibby et al. 1993); Diet and Trophic Ecology (DTE): these studies exclusively observe the feeding preferences of the species, stomach content, pellets, trophic position, trophic chains, and feeding activity; Reproduction and Development (RD): studies related to reproductive characteristics, courting, fertilization, nesting, hatching, number of hatchlings, waiting period in the nest, embryo development, and yearly development period; Ethology, Migration, and Home range (EMH): studies on migratory behavior, patterns, and habits, and home range according to the definition by Burt (1943) as the area occupied by an individual during feeding, mating, and nestling care; Parasitology and Medicine (PM): studies related to the internal or external parasitic flora of the species, new parasite species registries, clinical data, medical reports, and veterinary procedures; Conservation and Legislation (CL): studies regarding conservation plans, conservation state, anthropogenic effects, environmental legislation, hunting manuals, and hunting and closed-season reports; Study Methods and Techniques (SMT): studies on sample recollection and manipulation, notes on observation and data registry, and comparisons between study methods; and “Environmental Education and Science Outreach” (ESO): studies that focus on teaching environmental sciences by using birds of prey to a non-specialists audience.

Publications which did not explicitly mention the scientific or common name(s) of the study species were omitted from our data set.

## Results

**Figure 1.**
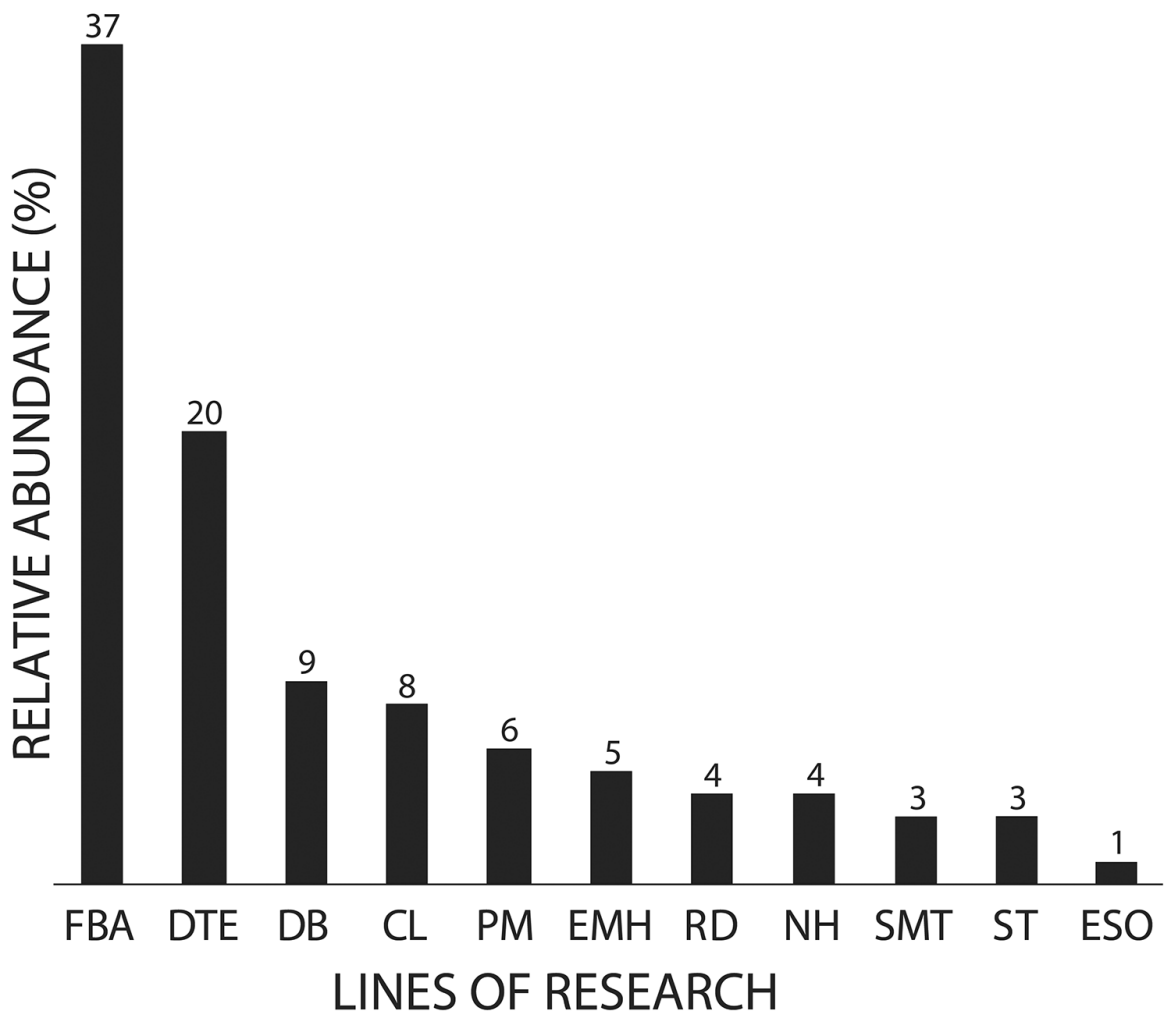
Relationship between different lines of investigation: (FBA) Faunistics, Biodiversity, and Abundance, (DTE) Diet and Trophic Ecology, (DB) Distribution and Biogeography, (CL) Conservation and Legislation, (PM) Parasitology and Medicine, (EMH) Ethology, Migration, and Home environment, (RD) Reproduction and Development, (NH) Natural History, (SMT) Study Methods and Techniques, (ST) Systematics and Taxonomy, and (ESO) Environmental Education and Science Outreach.

**Figure 2.**
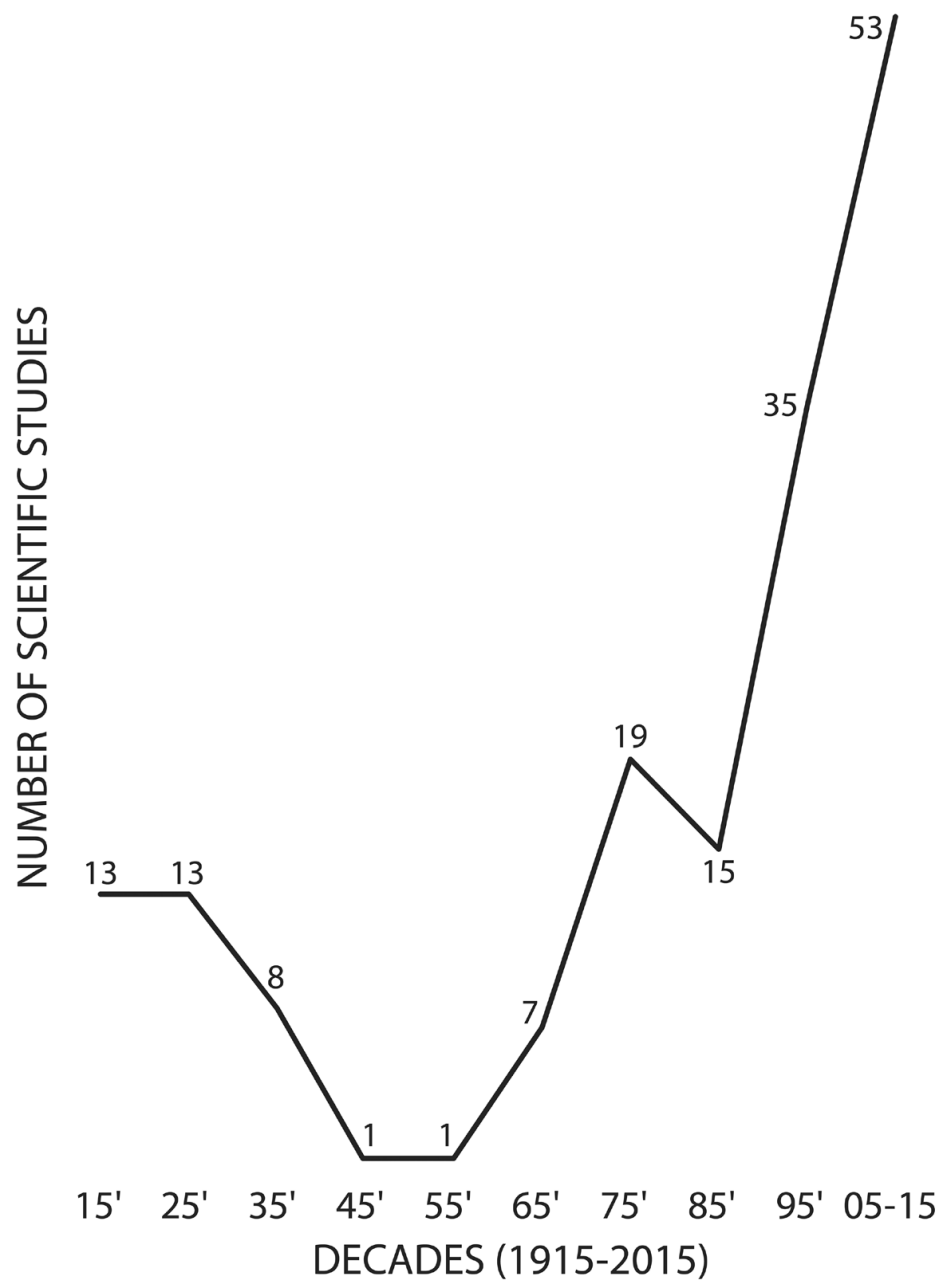
Number of studies regarding the Falconidae family published in the last century (from 1915 to 2015).

**Figure 3.**
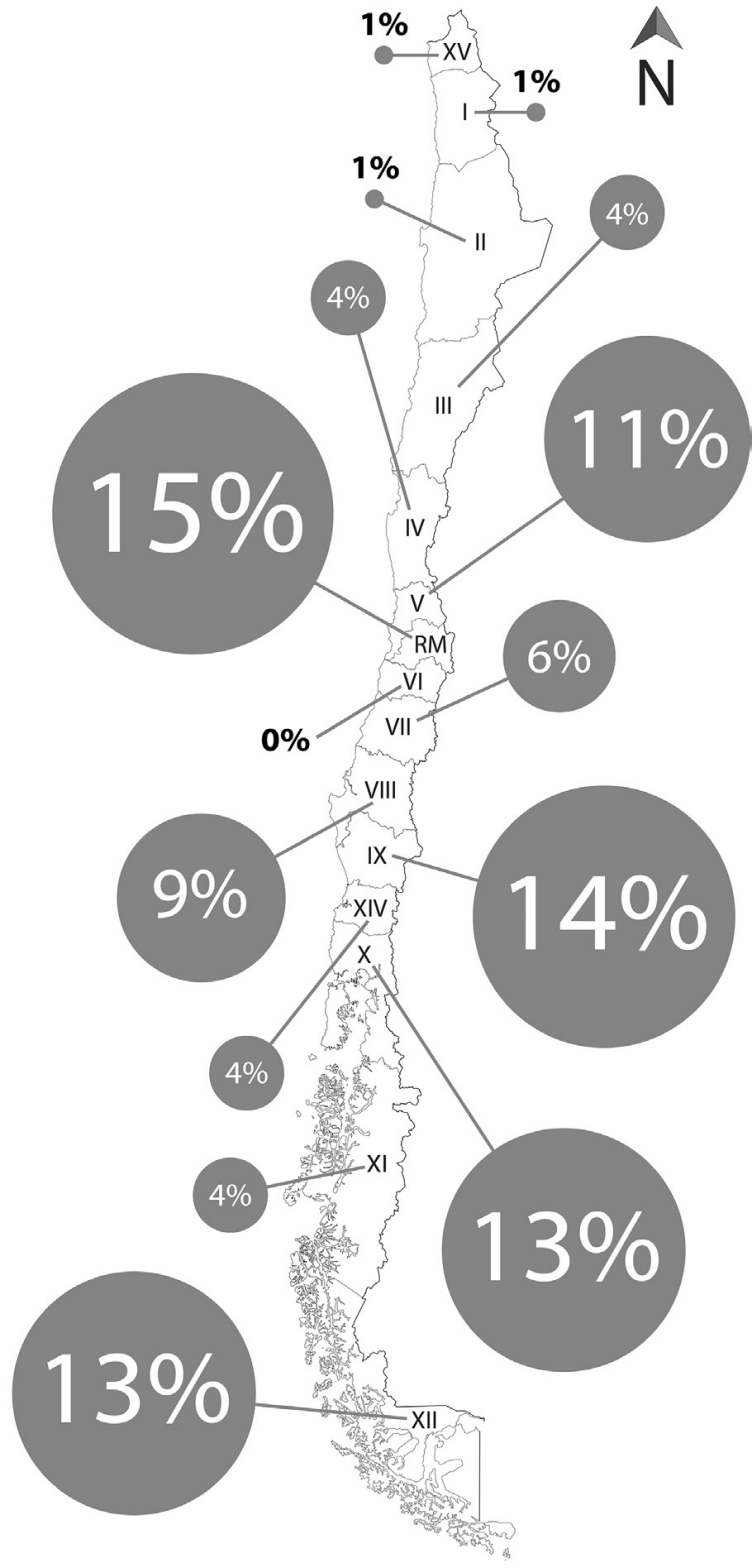
Relative abundance of studies published for each administrative region of Chile.

**Figure 4.**
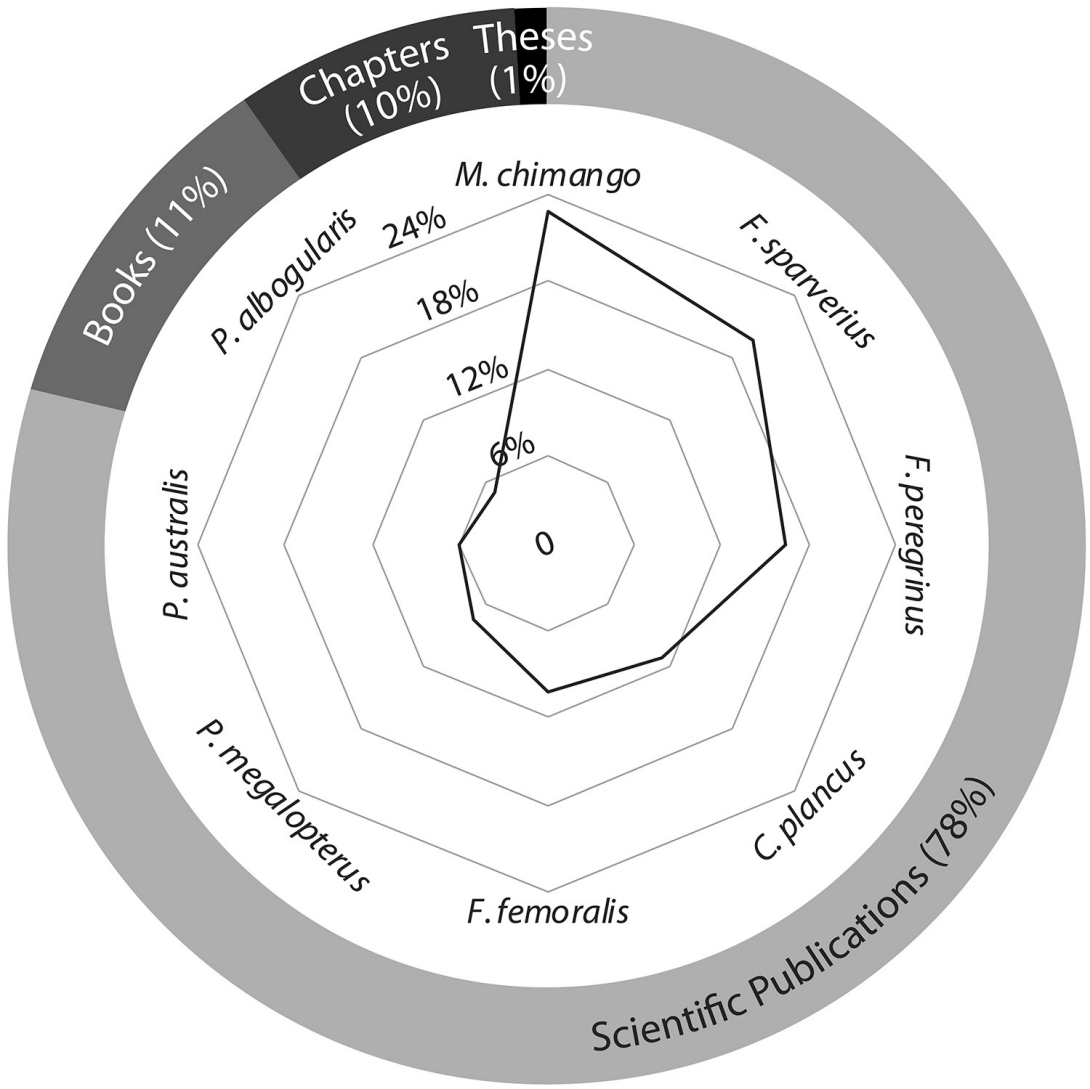
Relative bibliographic importance according to circulation medium and researched species.

A total of 165 studies between 1915 and 2015 included scientific data on falcons in Chile. The most studied research topics were “Faunistics, Biodiversity, and Abundance” and “Diet and Trophic Ecology” with 63 and 34 studies, respectively. “Study Methods and Techniques” and “Environmental Education and Science Outreach” were the least studied topics, with 5 and 1 publications, respectively (Figure 1). The highest productivity was observed during the last 10 years (2005–2015) with 52 publications, in contrast with the decade between 1945 and 1955 which only presented one study (Figure 2). Of the administrative regions (Figure 3), Metropolitan Santiago was best represented with 15% of contributions, followed by Araucanía with 14% and Los Lagos and Magallanes with 13% each. The O’Higgins region contributed no studies. Most (78%) studies were scientific papers, followed by books (11%), and books chapters (10%). Only 1% of publications were theses. Across species, *Milvago chimango*, *Falco sparverius*, and *Falco pererinus* were most often mentioned, with 23, 23, and 20% of contributions, respectively. The remaining species contributed fewer than 11% of citations (Figure 4).

In the following section, results are summarized for each one of the research topics:

*Natural history*. The natural history of falcons in Chile is poorly documented. Studies by Quijada (1917), Housse (1934; 1937), and Barros (1960) regarding *Milvago chimango*, by Housse (1935) regarding *Falco sparverius*, and Housse (1936a) regarding *Caracara plancus* discuss aspects of morphology, behaviour, reproduction, development, foraging, and as well as anecdotal observations of daily activity. Particularly, the complete reproductive cycle of *Phalcoboenus megalopterus* was described by Housse (1937), whereas data are missing for the remaining species. Figueroa (2015) analyzed the scarce literature regarding the natural history of *Milvago chimango*.

*Systematics and taxonomy*. The systematics and taxonomy of falcons have been clarified by Fuchs et al. (2012, 2015). They reported polyphyly of the genus *Milvago*, and found that *Milvago chimango* is part of the *Phalcoboenus* clade and should be transferred to this genus (Fuchs et al. 2012). The genus *Falco* was found to be monophyletic (Fuchs et al. 2015). There is little information regarding intraspecific variation and evolution of falcons in Chile. *Falco sparverius fernandensis* was described by Chapman (1915). Two subspecies of *Milvago chimanago* are known: *Milvago chimango temucoensis* (Sclater 1918) is found is most of Chile (Housse 1934) and *Milvago chimango fuegiensis* (Johnson and Behn 1957), which is endemic in Tierra del Fuego. The latter subspecies is no longer recognized as a valid taxon by most taxonomists (e.g. Dickinson and Remsen 2013). Drouilly’s (1968) identification key provides accurate diagnostic recognition characteristics for species classification. McNutt (1984) established that the pale coloration in *Falco peregrinus* is recessive in the genotype of the species.

*Distribution and biogeography*. The genera *Falco* (Kleinschmidt 1929; Reynolds 1934; Bullock 1949; Torres 1970; Araya et al. 1974; Schlatter 1976; Araya and Millie 1998), *Caracara* (Reynolds 1934), and *Milvago* (Jaksic et al. 2002) can be found from Arica to Magallanes regions. Among *Phalcoboenus*, *Phalcoboenus megalopterus* is the most widely distributed species occurring from the extreme north (Hellimayr 1932) south to Magallanes (Jaksic et al. 2002); *Phalcoboenus albogularis* (Vuilleumier 1985, Cursach et al. 2009) has been sighted once in the central area of Chile, and *Phalcoboenus australis* is limited to the extreme south (Reynolds 1934, Drouilly 1968, Collar 1986, Marín et al. 2006).

*Faunistics, biodiversity, and abundance*. During an extended period, only the presence or absence of species was documented without attention for abundance (e. g. Jaffuel and Pirion 1927, Gigoux 1928, Bullock 1929a, Bullock 1929b, Reynolds 1934, Housse 1936b, Philippi 1937, Barros 1937, Bullock 1938, Millie 1938, Larraín 1939, Araya et al. 1974, Cody 1970, Keith 1970). Population estimates began with general ornithofaunal observations at some locations, albeit in a non-systematic manner, which resulted in low estimates (with the exception of *Milvago chimango*) (e. g: Cody 1970, Markham 1970, Araya et al. 1974, Schlatter 1976, Ellis and Glinski 1980, Venegas 1981, Figueroa et al. 2000a, Simeone et al. 2008, Imberti 2005). The greatest abundance and number of species were registered in national parks and reserves (e.g. *Falco femoralis*, *Falco peregrinus*, and *Milvago chimango* in the National Ñuble Reserve, Estades 1997; *Caracara plancus*, *Falco femoralis*, and *Milvago chimango* in the Huemules de Niblinto Nature Sanctuary, Figueroa et al. 2000b; and *Caracara plancus*, *Falco peregrinus*, *Falco sparverius*, *Milvago chimango*, and *Phalcoboenus albogularis* in the National Futaleufú Reserve Elgueta et al. 2006).

*Diet and Trophic Ecology*. The feeding preferences and habits of *Caracara plancus*, *Falco femoralis*, *Falco sparverius* and *Milvago chimango* have been described (Barros 1925). *Falco femoralis* exclusively feeds on birds and insects (Jiménez 1993, Jaksic et al. 1993, 1996), depending on the food supply (Figueroa and Corales 2005). *Falco peregrinus* is considered an opportunist (McNutt 1981) and is a top predator of granivore birds (Marquet et al. 1998), as well as an avid insect eater, again depending on supply (Simeone et al. 1997). The feeding habits of *Falco sparverius* are known in detail; it is classified as a predator of insects, reptiles (Goodall et al. 1951, Jaksic et al. 1982, Jaksic and Ostfeld 1983, Marquet et al. 1998, Jaksic and Feinsinger 1991, Simeone et al. 1997), and small mammals (Jaksic 1986), and the supply of the latter increases species abundance (Jaksic et al. 1992, Jaksic et al. 1993, 1996). *Falco sparverius* is capable of changing its diet following the seasons (Figueroa and Corales 2002, Mella 2002, 2005) and geographic location (Ellis et al. 2002). No trophic superposition between *Falco femoralis* and *Falco sparverius* has been documented in Chile (Torres-Mura 2004, Rau and Jaksic 2004). The feeding habits and preferences of *Milvago chimango* are described in detail through stomach content and pellets (Yáñez et al. 1980): it is an omnivore and a scavenger (Núñez and Yáñez 1981, Núñez et al. 1982, Yáñez et al. 1982, Cabezas Schlatter 1987, Jaksic and Simonetti 1987, Sazima and Olmos 2009). Observations by Figueroa et al. (2004) reveal *Phalcoboenus megalopterus* as an insectivore and carnivore of small birds and mammals. Finally, *Phalcoboenus australis* has been described as the most important predator of *Eudyptes chrysocome* (Forster 1781) eggs (Sphenisciformes) (Cursach et al. 2014).

*Reproduction and Development*. Information on reproductive cycle and development is available for *Falco sparverius*, *Caracara plancus*, *Milvago chimango*, and *Phalcoboenus australis*. Nesting data on *Phalcoboenus megalopterus* in Chile are limited to a monograph by Housse (1937), who presumed the species would nest in the rich vegetation areas of the mountains. In general, falcons begin nesting in early spring, laying between 2 and 4 eggs, with an incubation period of 15 to 20 days (Housse 1934, Housse 1935, Housse 1936a, Morrison and Phillips 2000, Díaz and Armesto 2003, Marín et al. 2006). Raimilla et al. (2014) described cooperation in care and nursing in *Phalcoboenus australis*.

*Ethology, migration, and home environment*. Information for this topic is fragmented across a number of brief publications and reviews. *Milvago chimango* is a resident species associated with highly urbanized areas (Lobos et al. 2011). *Falco sparverius* and *Falco peregrinus* are classified as semi-residents (Teneb et al. 2013, Cursach and Rau 2008). The only migrant species of falcon recorded in Chile is *Falco femoralis* (Jaksic and Simonetti 1987). For instance, the species is considered only a summer visitor to Las Chinchillas National Reserve, North of Santiago (Jaksic and Lazo 1999).

*Parasitology and medicine*. Intestinal endoparasite fauna has been described for *Milvago chimango* (San Martín et al. 2006), and included bacteria (*Haemoproteus tinnunculi* (Wasielewski and Wilker 1918), *Leucocytozoon toddi* (Sambon 1908) (Protozoa: Haemosporine) (Forrester et al. 2001)), and ectoparasites (new Ischnocera species (Insecta: Phthiraptera) (Mey and González-Acuña 2000), and Argas (persicargas) keiransi (Acari: Argasidae)) sampled from the neck (Estrada-Peña et al. 2003). *Escherichia coli* (Migula 1985) has been identified as a cause of fatal meningoencephalitis in *Milvago chimango* (Seguel et al. 2012). The parasites described and registered in other species of falcons include *Degeeriella rufa* (Phthiraptera: Philopteridae) in *Falco femoralis* and *Falco peregrinus*; *Acutifrons megalopterus* (Carriker, 1956) (Phthiraptera: Ischnocera) and *Colpocephalum megalopteri* (Price 1967) (Phthiraptera: Menoponidae) in *Phalcoboenus megalopterus*; and *Laemobothrion tinnunculi* (Phthiraptera: Laemobothriidae) (González-Acuña et al. 2008) and nematodes, cestodes, and trematodes (Gonzáles-Acuña et al. 2011) in *Falco sparverius*.

*Conservation and legislation*. The only species of falcons in Chile which are considered to warrant conservation priority are *Phalcoboenus albogularis* and *Phalcoboenus australis* (Pavéz 2004, Trejo 2007). However, the mechanisms and processes that affect their population have not been identified. Population size of *Falco sparverius* and *Milvago chimango* in San Carlos de Apoquindo in the Andean foothills of Santiago has decreased over time (Pavéz et al. 2010). These species, along with *Falco peregrinus*, are at risk for population decrease due to anthropogenic factors (Díaz and Armesto 2003), including accidents such as electrocution on high-voltage lines (González et al. 2014). Tala and Iriarte (2004) provide important data regarding legislation and potential threats to falcons.

*Study methods and techniques*. Studies focusing on study methods and techniques are few. Nevertheless, much information is available on capture and monitoring (Pavéz 2004), diet (Yáñez et al. 1980; Muñoz-Pedreros and Rau 2004), observation and counting techniques (Márquez et al. 2004), and use of baits (Contreras and González 2007).

*Environmental education and science outreach*. The single paper on this subject (Figueroa 1995) provides a plan for teaching ecology and environmental biology through the use of birds of prey, including *Milvago chimango* and *Falco sparverius* species.

## Discussion

The results presented here show that knowledge on falcons in Chile is uneven across species, research topics and administrative regions. Despite the limitations of this type of study, our data have the advantage of directly visualizing gaps in knowledge of specific topics (Bimrose et al. 2005). Previous reviews (e. g. Muñoz-Pedreros and Norambuena 2011, Raimilla et al. 2012) have demonstrated a recent increase in productivity, mostly resulting from the work of dedicated researchers (Bierregaard 1995).

Given that the classification of research topics used were those of Muñoz-Pedreros and Norambuena (2011), it is not surprising that our review finds similar trends in productivity and circulation medium. Comparison to the study by Raimilla et al. (2012) is hampered by the fact that their findings were not reported separately by species and administrative region. Nevertheless, two clear patterns emerge from these two reviews and the present study: productivity has increased considerably during the last 30 years for birds of prey in general and for Falconidae in particular, and peer-reviewed publications are the most frequently-used circulation medium to report study results. These patterns are not exclusive to these groups but represent a general trend in all sciences (Bornmann and Mutz 2015).

Our review shows that the most common research topic in studies of falcons is “Faunistics, Diversity, and Abundance” and that *Milvago chimango* is the most frequent study species. In contrast, Raimilla et al. (2012) found that, studies focusing on “diet” were the most common study subject and *Falco sparverius* was the most commonly studied species of falcon. Population trends in time are hampered by the fact that older studies typically represented reports of the presence of species and provided no data on abundance. In contrast to Raimilla et al. (2012), we found that studies of *Milvago chimango* outnumbered those of *Falco sparverius*. However, in agreement with Raimilla et al. (2012) we found that members of the genus *Phalcoboenus* are poorly covered in the literature, as underscored by the absence of studies on four of the ten study topics.

The concentration of publications on falcons in a small number of regions (Metropolitan Santiago, Araucanía, Los Lagos, and Magallanes) reflects the location of research centers (Raimilla et al. 2012). The same pattern was observed in works that focus on community structure (Jaksic 1985) and continental water birds (Victoriano et al. 2006). There were no studies which report on falcons in the O'Higgins region (central Chile) despite its proximity to the Metropolitan region. The lack of studies may be due to changes in regional administration throughout time (Errázuriz 1998) that may lead to incorrect registry of avifauna near the political-geographic limits. Future studies should focus on poorly studied areas, especially those within a biodiversity hotspot such as Los Ríos and Aysén regions (Arroyo et al. 2008). Our review indicates that some topics remain little studied. Publications on “Study Techniques and Methods” were rare (five citations). We suggest these topics warrant further study because current studies are carried out based on protocols developed for falcons in the northern hemisphere (e.g. Bird and Bildstein 2007) which may not be optimal for research in Chile. The lack of knowledge on the biology of species native to Chile may hamper the application of techniques developed elsewhere for other species. The use of non-suitable techniques may produce biases or errors and perhaps even harm captured individuals (Ford 2003).

Only a single study dealt with “environmental education and divulgation” of falcons (Figueroa 1995). Other works have presented a complete bird of prey education program, but mainly focused on birds of prey in general (e.g. Möler and Muñoz and Pedreros 2004). Nevertheless, both studies illustrate how birds of prey (including falcons) may be used to increase awareness of various environmental problems to a non-specialized audience.

Knowledge on “natural History” is deficient for most species of falcons in Chile. Figueroa (2015) suggested that the lack of knowledge on natural history of *Milvago chimango* results from historical factors, a shift from field biology to other types of study, difficulties in studying diet, and perhaps changing interests of researchers. These factors may also apply to the study of other species of falcons in Chile.

## Conclusions

This review of the state of knowledge on falcons in Chile indicates bias in all assessed categories: research topics are biased towards faunistic data; geographically, with many subsampled or unsampled regions; and in preferred species, with two major researched species. Due to the increase in scientific productivity for Falconidae and birds of prey, especially in recent years, reporting where information is lacking and which regions and species have not been studied is essential to continue the study of this group optimally. Much remains to be done and further research is needed. We hope this work may provide an impetus to fill the historical, ecological, and geographical gaps in knowledge.

## References

[B1] Araya B Millie G (1998) Guía de campo de las aves de Chile. 8va Edición. Editorial Universitaria, Santiago, 406 pp.

[B2] Araya B Millie G Magnere O (1974) Aves del Parque Nacion al “Vicente Pérez Rosales”. Anales del Museo de Historia Natural de Valparaíso Chile 7: 311–316.

[B3] Arroyo M Marquet P Marticorena C Simonetti J Cavieres L Squeo F Rozzi R Massardo F (2008) El hotspot chileno, prioridad mundial para la conservación. In: Conama (Ed.) Biodiversidad de Chile, Patrimonios y Desafíos. 1ra Edición. Ocho Libros Editores, Santiago, Chile, 90–95.

[B4] Barros R (1920) Aves del valle de Nilahue (Segunda parte). Revista Chilena de Historia Natural 24: 43–49.

[B5] Barros R (1925) Observaciones ornitológicas relacionadas con la agricultura y la caza. Revista Chilena de Historia Natural 29: 238–279.

[B6] Barros R (1937) Aves observadas en Maullín. Revista Chilena de Historia Natural 41: 182–186.

[B7] Barros R (1960) El tiuque *Milvago chimango chimango* (Vieillot). Revista Universitaria 44/45: 31–37.

[B8] Bibby C Burgess N Hill D (1993) Bird Census Techniques. Academic Press, Cambridge, 257 pp.

[B9] Bierregaard R (1995) The biology and conservation status of Central and South American Falconiformes: a survey of current knowledge. Bird Conservation International 5: 325–340. https://doi.org/10.1017/S0959270900001076

[B10] Biondi L Bó M (2008) Experimental assessment of problem solving by *Milvago chimango* (Aves: Falconiformes). Journal of Ethology 26: 113–118. https://doi.org/10.1007/s10164-007-0035-2

[B11] Bimrose J Barnes S Brown J (2005) A systematic literature review of research into carreer Interventions for Higher Education. Institute for Employment Research University of Warwick, Warwick, 77 pp.

[B12] Bird D Bildstein K (2007) Raptor research and management techniques. Institute for Wildlife Research, National Wildlife Federation, Washington DC, 464 pp.

[B13] Bornmann L Mutz R (2015) Growth rates of modern science: A bibliometric analysis based on the number of publications and cited references. Journal of the Association for Information Science and Technology 66(1): 2215–2222. https://doi.org/10.1002/asi.23329

[B14] Brooke de L Hanley S Laughlin B (1999) The scaling of eye size with body mass in birds. Proceedings of the Royal Society 266: 405–412. https://doi.org/10.1098/rspb.1999.0652

[B15] Bullock D (1929a) Aves observadas en los alrededores de Angol. Revista Chilena de Historia Natural 33: 171–211.

[B16] Bullock D (1929b) Aves de los pinares de Nahuelbuta. Revista Chilena de Historia Natural 33: 121–127.

[B17] Bullock D (1932) Los nombres científicos de Molina. Revista Chilena de Historia Natural 36: 113–117

[B18] Bullock D (1938) Aves observadas en la Región de Toltén. Revista Chilena de Historia Natural 42: 105–114.

[B19] Bullock D (1949) Sobre algunas aves norteamericanas en Chile. Boletín de la Sociedad de Biología de Concepción 24: 7–14.

[B20] Burt W (1943) Territoriality and home range concepts as applied to mammals. Journal Mammal 24: 346–352. https://doi.org/10.2307/1374834

[B21] Cabezas V Schlatter R (1987) Hábitos y comportamientos alimentarios de *Milvago chimango* Vieillot Aves: Falconidae. Anales del Museo de Historia Natural de Valparaíso Chile 18: 131–141.

[B22] Chapman F (1915) Descriptions of New Birds from Colombia, Ecuador, Peru, and Argentina. American Museum Novitates 80: 515–516.

[B23] Cody M (1970) Chilean bird distribution. Ecology 51: 455–464. https://doi.org/10.2307/1935380

[B24] Collar N (1986) Threatened Raptors of Americas: Work in Progress from the ICBP/IUCN Red Data Book. Bird of Prey Bulletin 3: 13–25.

[B25] Contreras P González M (2007) Respuestas en aves rapaces frente al uso de señuelos acústicos en censos diurnos y nocturnos en el sur de Chile. Gestión Ambiental 14: 79–87.

[B26] Cory B (1915) Notes on South American birds, with descriptions of new subespecies. Field Museum of Natural History, Chicago, 48 pp https://doi.org/10.5962/bhl.title.2570

[B27] Cursach J Rau J (2008) Avifauna presente en dos parques urbanos de la ciudad de Osorno, sur de Chile. Boletín Chileno de Ornitología Chile 14: 98–103.

[B28] Cursach J Suazo C Rau J Niklitschek E Vilugrón J (2014) Observaciones sobre el pingüino de penacho amarillo *Eudyptes c. chrysocome* en isla Gonzalo, Archipiélago Diego Ramírez, Chile. Revista de Biología Marina y Oceanografía 49: 361–366.

[B29] Díaz I Armesto J (2003) La conservación de las aves silvestres en los ambientes urbanos de Santiago. Ambiente y Desarrollo 19: 31–38.

[B30] Dickinson EC Remsen JV Jr (2013) The Howard and Moore complete checklist of the birds of the world. Fourth edition, vol 1: Non-passerines. Aves Press, London, 461 pp.

[B31] Drouilly P (1968) Clave de identificación de los Falconiformes de Chile. Noticiario Mensual Museo de Historia Natural 12: 3–10.

[B32] Elgueta E Reid S Pliscoff P Méndez M Núnez J Smith-Ramírez C (2006) Catastro de vertebrados terrestres y análisis en seis hábitats presentes en la Reserva Nacional Futaleufú, Provincia de Palena, X Región, Chile. Gayana 70: 195–205. https://doi.org/10.4067/s0717-65382006000200006

[B33] Ellis D Glinski R (1980) Some unusual records for the Peregrine and Pallid falcons in South America. Condor 82: 350–351. https://doi.org/10.2307/1367410

[B34] Ellis D Sabo B Fackler J Millsap B (2002) Prey of the peregrine falcon (*Falco peregrinus casssini*) in southern Argentina and Chile. Journal of Raptor Research 36: 315–320.

[B35] Estades C (1997) Bird habitat relationships in a vegetational gradient in the Andes of central Chile. Cóndor 99: 719–727. https://doi.org/10.2307/1370483

[B36] Errázuriz A (1998) Manual de Geografía de Chile. Editorial Andrés Bello, Santiago.

[B37] Estrada-Peña A Venzal J González-Acuña D Guglielmone A (2003) Argas (Persicargas) keiransi n. sp. (Acari: Argasidae), a parasite of the Chimango, *Milvago c. chimango* (Aves: Falconiformes) in Chile. Morphology Systematics and Evolution 40: 766–769. 10.1603/0022-2585-40.6.766 14765651

[B38] Figueroa R (2015) El rapaz olvidado – ¿Por qué hay tan pocos estudios sobre la historia natural y ecología básica del tiuque (*Milvago chimango*) en Chile? Boletin Chileno de Ornitología 21(1–2): 103–118.

[B39] Figueroa R Alvarado S Corales E González-Acuña D Schlatter R Martínez D (2015) Los Búhos de Chile. In: Enríquez P (Ed.) Los búhos Neotropicales, Diversidad y Conservación. El Colegio de la Frontera Sur (ECOSUR), San Cristóbal de las Casas, Chiapas, México Primera Edición, 173–273.

[B40] Figueroa R Corales E (2002) Winter diet of the American Kestrel (*Falco sparverius*) in the forested Chilean Patagonia, and its relation to the availability of prey. International Hawkwatcher 5: 7–14.

[B41] Figueroa R Corales E (2005) Aplomado Falcon (*Falco femoralis*) in an agricultural area of araucanía, southern Chile. Journal of Raptor Research 39: 55–60.

[B42] Figueroa R Orellana A Corales E (2004) Notes on a range expansion and summer diet of the mountain Caracara in the Andes of South-Central Chile. Journal of Raptor Research 28(3): 290–292.

[B43] Figueroa R Corales E Cerda J Saldivia H (2000a) Roedores, rapaces y carnívoros de Aysén. Servicio Agrícola y Ganadero, Gobierno Regional de Aysén, 195 pp.

[B44] Figueroa R Bravo C Corales E López R Alvarado S (2000b) Avifauna del Santuario de la Naturaleza Los Huemules del Niblinto, Región del Bío Bío, Chile. Boletín Chileno de Ornitología 7: 2–12.

[B45] Ford S (2003) Avian Care and Handling at the Alaska Raptor Center. Reference materials for volunteers in the performance of husbandry, bird training, and basic medical care. Alaska Raptor Center, 132 pp.

[B46] Forrester D Foster G Morrison J (2001) *Leucocytozoon toddi* and *Haemoproteus tinnunculi* (Protozoa: Haemosporina) in the chimango caracara (*Milvago chimango*) in southern Chile. Memorias do Instituto Oswaldo Cruz 96: 1023–1024. 11685273 10.1590/s0074-02762001000700024

[B47] Fuchs J Johnson J Mindell D (2012) Molecular systematic of the caracaras and allies (Falconidae: Polyborinae) inferred from mitochondrial and nuclear sequence data. Ibis 154: 520–532. https://doi.org/10.1111/j.1474-919X.2012.01222.x

[B48] Fuchs J Johnson JA Mindell DP (2015) Rapid diversification of falcons (Aves: Falconidae) due to expansion of open habitats in the Late Miocene. Molecular Phylogenetics and Evolution 82: 166–182. https://doi.org/10.1016/j.ympev.2014.08.010 25256056 10.1016/j.ympev.2014.08.010

[B49] Gigoux E (1928) Aves de la quebrada del León y alrededores. Revista Chilena de Historia Natural 32: 144–148.

[B50] González G Ossa G Sánchez L Silva R (2014) Medidas de mitigación de impactos en aves silvestres y murciélagos. Análisis de Información encargado por el SAG (Servicio Agrícola y Ganadero), Ministerio del Medio Ambiente, Santiago, 984 pp.

[B51] González-Acuña D Ardiles K Figueroa R Barrientos C González P Moreno L Cicchin A (2008) Lice of Chilean diurnal raptors. Journal of Raptor Research 42: 281–286. https://doi.org/10.3356/JRR-07-69.1

[B52] González-Acuña D Lohse E Cicchino A Mironov S Figueroa R Ardiles K Kinsella M (2011) Parasites of the American Kestrel *Falco sparverius* in south-Central Chile. Journal of Raptor Research 45: 188–193. https://doi.org/10.3356/JRR-10-68.1

[B53] Goodall J Johnson A Philippi R (1951) Las aves de Chile, su conocimiento y sus costumbres. Vol. 2. Platt Establecimientos Gráficos S. A, Buenos Aires, Argentina, 445 pp.

[B54] Hellmayr C (1932) Birds of Chile. Field Museum of Natural History Publications 308 (Zoological Series) 19: 1–472.

[B55] Housse R (1934) Monografía del tiuque. Revista Chilena de Historia Natural 38: 49–53.

[B56] Housse R (1935) Monografía del cernícalo *Cerchneis sparveria cinnamemina* (Swainson). Revista Chilena de Historia Natural 39: 59–63.

[B57] Housse R (1936a) Monografía del traro *Polyborus plancus plancus* (Miller). Revista Chilena de Historia Natural 40: 19–26.

[B58] Housse R (1936b) Avifauna de la Isla Santa María. Revista Chilena de Historia Natural 40: 63–69.

[B59] Housse R (1937) El tiuque Cordillerano, *Phalcoboenus megalopterus*. Revista Chilena de Historia Natural 41: 131–134.

[B60] Imberti S (2005) Distribución otoñal de aves marinas y terrestres en los canales Chilenos. Anales del Instituto de la Patagonia 33: 21–30.

[B61] Jaffuel F Pirion A (1927) Aves observadas en el valle de Marga-Marga. Revista Chilena de Historia Natural 31: 102–115.

[B62] Jaksic F (1986) Predation upon small mammals in shrublands and grasslands of southern South America: ecological correlates and presumable consequences. Revista Chilena de Historia Natural 59: 209–221.

[B63] Jaksic F Feinsinger P (1991) Bird assemblages in temperate forests of North and South America: a comparison of diversity, dynamics, guild structure and resource use. Revista Chilena de Historia Natural 64: 491–510.

[B64] Jaksic FM Lazo I (1999) Response of a bird assemblage in semiarid Chile to the 1997–1998 El Niño. Wilson Bulletin 111: 527–535.

[B65] Jaksic F Simonetti J (1987) Predator/prey relationships among terrestrial vertebrates: an exhaustive review of studies conducted in southern South America. Revista Chilena de Historia Natural 60: 221–244.

[B66] Jaksic F Ostfeld R (1983) Numerical and behavioral estimates of predation upon rabbits in Mediterranean type shrublands: a paradoxical case. Revista Chilena de Historia Natural 56: 39–49.

[B67] Jaksic F Greene H Schwenk K Seib R (1982) Predation upon reptiles in Mediterranean habitats of Chile, Spain and California: a comparative analysis. Oecologia 53: 152–159. https://doi.org/10.1007/BF00545658 10.1007/BF00545658 28311104

[B68] Jaksic F Iriarte J Jiménez J (2002) The raptors of Torres del Paine National Park: species accounts, diversity, and niche relationships. Revista Chilena de Historia Natural 75: 449–461. https://doi.org/10.4067/S0716-078X2002000200014

[B69] Jaksic F Jiménez J Castro S Feinsinger P (1992) Numerical and functional response of predators to a long-term decline in mammalian prey at a semiarid Neotropical site. Oecologia 89: 90–101. https://doi.org/10.1007/BF00319020 10.1007/BF00319020 28313400

[B70] Jaksic F Feinsinger P Jiménez J (1993) A long-term study of the dynamics of guild structure among predatory vertebrates at a semi-arid Neotropical site. Oikos 67: 87–96. https://doi.org/10.2307/3545099

[B71] Jaksic F Feinsinger P Jiménez J (1996) Ecological redundancy and long-term dynamics of vertebrate predators in semiarid Chile. Conservation Biology 10: 252–262. https://doi.org/10.1046/j.1523-1739.1996.10010252.x

[B72] Jaksic F Pavéz E Jiménez J Torres-Mura J (2001) The conservation status of raptor in the Metropolitan Region, Chile. Journal of Raptor Research 35: 151–158.

[B73] Jiménez J (1993) Notes of the diet of the Aplomado falcon (*Falco femoralis*) in northcentral Chile. Journal of Raptor Research 27: 161–163.

[B74] Keith A (1970) Bird Observation from Tierra del Fuego. The Condor 72(3): 361–363. https://doi.org/10.2307/1366015

[B75] Kleinschmidt O (1929) *Falco kreyenborgi*. Falco 3: 33–35.

[B76] Larraín T (1939) Sobre las aves de rapiña observadas en la hacienda “San Jerónimo”, zona de Casablanca, vecina la mar, provincia de Valparaíso. Revista Chilena de Historia Natural, 43: 116–123.

[B77] Lobos G Bobdilla P Alzamora A Thomson R (2011) Patrón de actividad y abundancia de aves en un relleno sanitario de Chile central. Revista Chilena de Historia Natural 84: 107–113. https://doi.org/10.4067/S0716-078X2011000100008

[B78] Marín M Kusch A Oehler D Drieschman S (2006) Distribution, breeding and status of the striated Caracara *Phalcoboenus australis* (Gmelin, 1788) in southern Chile. Anales del Instituto de la Patagonia (Chile) 34: 65–74.

[B79] Markham B (1970) Reconocimiento faunístico del área de los fiordos Toro y Cóndor, Isla Riesco, Magallanes. Anales del Instituto de la Patagonia Chile 11: 41–59.

[B80] Márquez C Sánchez I Rau J (2004) Técnicas de observación y estimación de abundancia de aves rapaces. In: Muñoz-Pedreros A Rau Yáñez J (Eds) Aves Rapaces de Chile. CEA Ediciones, Valdivia, 253–264.

[B81] Marquet P Bozinovic F Bradshaw G Cornelius C Gonzalez H Gutiérrez J Hajek E Lagos J Lopez-Cortez F Núñez L Rosello E Santoro C Samaniego H Standen V Torres-Mura J Jaksic F (1998) Los ecosistemas del desierto de Atacama y área andina adyacente en el norte de Chile. Revista Chilena de Historia Natural 71: 593–617.

[B82] McNutt J (1981) Selección de presa y comportamiento de caza del Halcón peregrino (*Falco peregrinus*) en Magallanes y Tierra del Fuego. Anales del Instituto de la Patagonia 12: 221–228.

[B83] McNutt J (1984) A Peregrine falcon polymorph: observation of the reproductive behavior of *Falco kreyenborgi*. The Condor 86: 378–382. https://doi.org/10.2307/1366810

[B84] Mella J (2002) Dieta del Cernícalo *Falco sparverius* y del Tucúquere *Bubo magellanicus* en un ambiente cordillerano de Chile central. Boletín Chileno de Ornitología 9: 34–37.

[B85] Mella J (2005) Cambios estacionales en la avifauna del Monumento Natural El Morado, cordillera de Santiago. Boletín Chileno de Ornitología 11: 2–10.

[B86] Méndez P Curti M Herrera de Montuto K Benedetti A (2006) Las aves Rapaces. Guía Didáctica de Educación Ambiental. The Peregrine Fund Editorial, Panamá, 124 pp.

[B87] Mey E González-Acuña D (2000) A new genus and species of Ichnocera (Insecta, Phthiraptera) of Chimango Caracara, *Milvago chimango* from Chile with annotated checklist off chewing lice parasitizing caracaras (Aves, Falconiformes, Falconidae). Rudolstäter Naturhistoriche Schriftent 10: 59–73.

[B88] Millie W (1938) Las aves del valle del Huasco y sus alrededores (Prov. de Atacama). Revista Chilena de Historia Natural 42: 181–205.

[B89] Moore E (1916) Excursión a la Península de Taitao. Boletín del Museo Nacional de Chile 9: 143–171.

[B90] Morrison J Phillips L (2000) Nesting habitat and success of the *Chimango Caracara* in southern Chile. Wilson Bulletin 112: 225–232. https://doi.org/10.1676/0043-5643(2000)112[0225:NHASOT]2.0.CO;2

[B91] Muñoz-Pedreros A Norambuena H (2011) Dos siglos de conocimiento de las aves rapaces de Chile (1810–2010). Gestión Ambiental 21: 69–93.

[B92] Muñoz-Pedreros A Rau J (2004) Estudio de egagrópilas en aves rapaces. In: Muñoz-Pedreros A Rau J Yáñez J (Eds) Aves Rapaces de Chile. CEA Ediciones, Valdivia.

[B93] Muñoz-Pedreros A Rau J Yáñez J (2004) Aves Rapaces de Chile. CEA Ediciones Valdivia.

[B94] Núñez H Yáñez J (1981) Alimentación del Tiuque *Milvago chimango* (Vieillot) (Aves: Falconiformes). Noticiario Mensual del Museo Nacional de Historia Natural Chile 25: 5–9.

[B95] Núñez H Sallaberry M Vergara R Yáñez J (1982) Alimentación anual de *Milvago chimango* (Vieillot) (Aves: Falconiformes). Boletín Museo Nacional de Historia Natural Chile 39: 125–130.

[B96] Pavéz E (2004) Descripción de las aves rapaces chilenas. In: Muñoz-Pedreros A Rau J Yáñez J (Eds) Aves Rapaces de Chile. CEA Ediciones, Valdivia, 29–104.

[B97] Pavéz E Lobos G Jaksic F (2010) Cambios de largo plazo en el paisaje y los ensambles de micromamíferos y rapaces en Chile central. Revista Chilena de Historia Natural 83: 99–111.

[B98] Philippi R (1937) Aves de la región de Zapallar. Revista Chilena de Historia Natural 41: 28–38.

[B99] Raimilla V Rau J Muñoz-Pedreros A (2012) Estado de arte del conocimiento de las aves rapaces de Chile: Situación actual y proyecciones futuras. Revista Chilena de Historia Natural 85: 469–480. https://doi.org/10.4067/S0716-078X2012000400009

[B100] Quijada B (1917) La ornitología chilena en el diccionario de la lengua castellana. Boletín del Museo Nacional de Chile 10: 5–27.

[B101] Raimilla V Suazo C Robertson G Rau J (2014) Observations suggesting cooperative breeding by Striated Caracaras (*Phalcoboenus australis*). Journal Raptor Research 48: 189–191. https://doi.org/10.3356/JRR-12-49.1

[B102] Rau J Jaksic F (2004) Diversidad de las aves rapaces de Chile. In: Muñoz-Pedreros A Rau J Yáñez J (Eds) Aves Rapaces de Chile. CEA Ediciones, Valdivia, 121–128.

[B103] Reynolds P (1934) Apuntes sobre aves de Tierra del Fuego. El Hornero 5: 339–353.

[B104] Rottmann J (1972) Algunas aves silvestres de los valles agrícolas inferiores a 1.000 m de altitud en el Departamento de Arica. IDESIA Chile 2: 59–63.

[B105] San Martín J Brevis I Rubilar C Krone O Gonzalez-Acuña D (2006) Parasitismo gastrointestinal en tiuque común *Milvago chimango chimango* (Vieillot, 1816) (Aves, Falconidae) en la zona de Ñuble, Chile. Parasitología Latinoamericana 61: 63–68. https://doi.org/10.4067/S0717-77122006000100009

[B106] Sazima I Olmos F (2009) The Chimango Caracara (*Milvago chimango*), an additional fisher among Caracarini falcons. Biota Neotropica Brasil 9: 403–405. https://doi.org/10.1590/S1676-06032009000300036

[B107] Schlatter R (1976) Aves observadas en el sector del Lago Riñihue, provincia de Valdivia, con alcances sobre su ecología. Boletín de la Sociedad de Biología de Concepción Chile 50: 133–143.

[B108] Seguel M Moroni M Gómez M Hernández C Paredes E (2012) Bacterial meningoencephalitis in a free Chimango Caracara (*Milvago chimango temucoensis*). Brazilian Journal of Veterinary Pathology 5: 16–19.

[B109] Simeone A Oviedo E Bernal M Flores M (2008) Las aves del Humedal de Mantagua: Riqueza de especies, amenazas y necesidades de conservación. Boletín Chileno de Ornitología 14: 22–35.

[B110] Simeone A Valencia J Schlatter R Lanfranco D Ide S (1997) Depredación de aves sobre larvas de *Rhyacionia buoliana* en plantaciones jóvenes de *Pinus radiata*. Bosque 182: 67–75. https://doi.org/10.4206/bosque.1997.v18n2-07

[B111] Tala C Iriarte A (2004) Conservación y Legislación. In: Muñoz-Pedreros A Rau J Yáñez J (Eds) Aves Rapaces de Chile. CEA Ediciones Valdivia, 281–294.

[B112] Teneb E Gomez H Cárcamo J (2013) Cronotipos en aves del Humedal de tres Puentes, Punta Arenas, Magallanes, Chile. Anales del Instituto de la Patagonia (Chile) 41: 61–69. https://doi.org/10.4067/S0718-686X2013000100005

[B113] Torres D (1970) Cernícalo *Falco sparverius fernandensis* (Chapman 1915) en Isla Alejandro Selkirk. Noticiario Mensual del Museo Nacional de Historia Natural Chile 14: 10.

[B114] Torres-Mura J (2004) Lista de aves rapaces de Chile. In: Muñoz-Pedreros A Rau J Yáñez J (Eds) Aves Rapaces de Chile. CEA Ediciones. Valdivia, 11–14.

[B115] Trejo A (2007) Identificación de especies y áreas prioritarias para el estudio de la reproducción de aves rapaces de Argentina. Hornero 22: 85–96.

[B116] Venegas C (1981) Aves de las Islas Wollaston y Bayly, Archipiélago del Cabo de Hornos. Anales del Instituto de la Patagonia Chile 12: 213–219.

[B117] Vázquez A Romero H (2007) Efectos ambientales de la Expansión Urbana de Alta y Baja Densidad en el Gran Santiago Durante las últimas tres Décadas. Anales de la Sociedad Chilena de Ciencias Geográficas 2007: 232–236.

[B118] Victoriano P González A Schlatter R (2006) Estado de conocimiento de las aves de aguas continentales de Chile. Gayana 70: 140–162. https://doi.org/10.4067/s0717-65382006000100019

[B119] Vuilleumier F (1985) Forest birds of Patagonia: ecological geography, speciation, endemism and faunal history. In: Buckley P Foster M Morton E Riedely R Buckley F (Eds) Neotropical Ornithology. Ornithological Monographs 36: 255–304. https://doi.org/10.2307/40168287

[B120] White C Olsen P Kiff L (1994) Family Falconidae (Falcons and Caracaras). In: del Hoyo J Elliott A Sargatal J (Eds) Handbook of the Birds of the World, Vol. 2 Lynx Ediciones, Barcelona, 216–275.

[B121] Yáñez JL Núñez H Jaksic FM (1980) Diet and weight of American Kestrels in central Chile. Auk 97: 629–631.

[B122] Yáñez JL Núñez H Jaksic FM (1982) Food habits and weight of Chimango Caracara in central Chile. Auk 99: 170–171. https://doi.org/10.2307/4086036

